# A Study of United States Registered Dietitian Nutritionists during COVID-19: From Impact to Adaptation

**DOI:** 10.3390/nu14040907

**Published:** 2022-02-21

**Authors:** Tracy L. Oliver, Rebecca Shenkman, Janell L. Mensinger, Caroline Moore, Lisa K. Diewald

**Affiliations:** 1 M. Louise Fitzpatrick College of Nursing, Villanova University, Villanova, PA 19085, USA; 2 MacDonald Center for Obesity Prevention and Education, Villanova University, Villanova, PA 19085, USA; rebecca.shenkman@villanova.edu (R.S.); lisa.diewald@villanova.edu (L.K.D.); 3 Department of Clinical and School Psychology, Nova Southeastern University, Fort Lauderdale, FL 33314, USA; jmensing@nova.edu; 4 Georgia Baptist College of Nursing, Mercer University, Atlanta, GA 30341, USA; moore_ch@mercer.edu

**Keywords:** pandemic, dietitians, lifestyle habits, weight change, eating patterns

## Abstract

The COVID-19 pandemic introduced an unprecedented health crisis, requiring many Registered Dietitian Nutritionists (RDNs) to expand their duties and services, while other RDNs faced unemployment, reduced hours, and changes to their work environment. This study evaluated whether the pandemic impacted RDNs’ weight, eating behaviors, and psychological factors, and whether professional training as an RDN was perceived as a protective factor in maintaining healthy habits. A 57-item, cross-sectional, online questionnaire including open-ended questions was distributed to RDNs residing in the United States. Over two months (January 2021 to February 2021), 477 RDNs completed the questionnaire. Among RDNs, 68.5% reported no weight change, 21.4% reported weight gain greater than 5 pounds, and 10.3% reported weight loss greater than 5 pounds. Approximately 75% (*n* = 360) reported their RDN professional training equipped them with the skills needed to maintain healthy eating behaviors. Reduced physical activity and mental health were the top qualitative themes that emerged regarding reasons for weight change. These findings suggest that RDN professional practice skills may have conferred some personal health benefits, as evidenced by smaller weight gains, the maintenance of healthy habits, and fewer reporting psychological effects relative to the general population and other health professionals, thereby limiting the impact of pandemic-induced work and life disruptions.

## 1. Introduction

The COVID-19 pandemic triggered a global health crisis that prompted a cascade of abrupt life and work adaptations with numerous health and professional consequences. While it is well documented that stressful life events can cause a variety of health effects such as shifts in eating patterns, diet quality, physical activity, and mental health, research continues to build on and explore the immediate and far-reaching impact of COVID-induced home, work, and overall lifestyle disruptions [1,2,3,4,5,6,7,8]. Notably, healthcare workers have been identified as a hard-hit segment of the workforce, susceptible to physical and psychological health consequences, i.e., increased levels of stress, anxiety, and emotional fatigue due to job-related stress [1,2,9]. 

Forced to step out of their comfort zones to face new daily challenges, healthcare workers have experienced and have been impacted by the COVID-19 pandemic in numerous ways. Some healthcare workers have been thrust onto the frontlines of care while placing themselves at risk for contracting the COVID-19 virus. Specifically looking at the role of registered dietitian nutritionists (RDNs) during the pandemic, many RDNs have been expected to take on additional responsibilities as credentialed practitioners, including the screening, treatment, and care of COVID-19 patients [10]. Despite the expanding roles of some RDNs, many others faced unemployment, reduced hours, changes to the work environment, working from home, and transitioning to a telehealth-based practice, all of which burdened some with additional psychological stressors and financial hardships [11,12,13]. One study conducted on RDNs outside the U.S. indicated that perceptions of work-related well-being (factors such as working remotely, changes in employment status, or financial concerns) significantly worsened during the pandemic [11].

Compromised well-being at work has been associated with maladaptive psychological consequences such as heightened anxiety and stress, as well as unhealthy coping practices such as smoking, drinking, overeating, and lack of exercise [2,11]. Stressful life events can affect health in a variety of ways, such as behavioral shifts in eating patterns, reduced diet quality, increased alcohol abuse, and unintentional weight change. Stress and ineffective coping mechanisms are also frequently associated with the increased consumption of less nutritive but highly palatable “comfort foods” that are typically energy-dense and high in sugar and fat [1,14,15]. Since work plays an essential and central role for many, significant changes, challenges, and uncertainties in life can drastically impact mental and physical well-being.

Despite growing attention to the pandemic’s impact on the well-being of healthcare workers, no study to date in the United States or worldwide has identified and described the pandemic experience—from impact to adaptation—from RDNs’ own perspectives. RDNs are nutrition experts who play an integral part in maintaining the health of individuals and communities, but stressful circumstances can present challenges to even those who are well-equipped with a strong foundation in nutritional knowledge. Studies have identified barriers to nutrition care practice and reported on changes in the work environment as well as resilience levels of dietitians during the COVID-19 pandemic. However, research evaluating the impact of the pandemic on the personal health of dietitians is lacking [16,17]. This present study seeks to represent RDNs in emerging research and uncover new information on the influence of stress and life disruption on these professionals’ weight, health habits, psychological factors, and professional practices, and on whether professional training as an RDN can be perceived as a protective factor in maintaining healthy habits.

## 2. Materials and Methods

### 2.1. Study Group

This exploratory and cross-sectional study investigated changes in health habits, lifestyle, psychological factors, and professional practice among RDNs during the COVID-19 pandemic. RDNs residing in the United States were recruited using a pre-existing electronic listserv of 4984 RDNs from January 2021 to February 2021. These months represented a time in the United States when new COVID cases peaked, and many parts of the country saw its highest rates since the beginning of the pandemic. Eligible participants were invited to complete a 57-item questionnaire anonymously through the Qualtrics^xm^ LLC system [18]. Interested participants provided informed consent electronically. In addition, optional open-ended questions obtained information regarding reasons for weight change, employment status, and lifestyle changes. One open-ended question was qualitatively analyzed (Q1), with another open-ended question (Q2) inviting participants to share additional context to responses to Q1.

### 2.2. Ethical Approval

This cross-sectional research methodology included an online questionnaire. All subjects participating in the project were informed of the aims and type of research and completed electronic consent to participate in the survey. Ethical review and approval were granted for this study from the Villanova University Institutional Review Board (Approval IRB-FY2021-139). The study was conducted in accordance with the Declaration of Helsinki.

### 2.3. Measures

#### 2.3.1. Demographic, Weight, Eating Patterns, Physical Activity, and Psychological Factors

Participants completed quantitative self-report measures about eating patterns, physical activity, and weight changes, along with demographic characteristics, employment status, professional practice setting, and years in professional practice. The research team developed these questions. Psychological factors were assessed using validated scales such as General Anxiety Disorder GAD-7 [19] and Insomnia Severity Index (ISI) [20]. Additionally, questions about the impact of COVID-19 on caregiver roles or professional responsibilities were included. 

#### 2.3.2. Qualitative Questions

Qualitative questions provided a unique opportunity to understand personal aspects of the pandemic experience. For example, the qualitative question Q1 explored reasons for changes in weight during the COVID-19 pandemic by asking, “If your weight changed during the height of the pandemic period, please describe why you think your weight changed.” Qualitative question Q2 provided a rich perspective to Q1 by asking, “If you would like, feel free to share more about how the COVID-19 pandemic affected your eating habits, food purchasing habits, physical activity, weight or general health.”

### 2.4. Data Coding and Analysis

Researchers exported data from Qualtrics [18] into SPSS v24.0 [21] for data analysis. Descriptive statistics (means and standard deviations) were computed to summarize the data. Additionally, frequencies and proportions were generated to characterize the sample. Self-reported weight change was calculated from weight prior to the pandemic and weight at the height of the pandemic. The GAD-7 scores for anxiety symptoms were calculated based on validation population norms shown in Lowe et al. [22]. In accordance with Morin et al. [23], ISI categories reflecting the severity of insomnia were computed.

Q1 was analyzed using AtlasTi9 with a content analysis approach [24,25]. Four research team members coded data to identify themes that emerged from individual responses. Next, the qualitative responses were read and independently coded by each research team member. Following discussions, preliminary themes were shared, compared to address discrepancies, and finalized. Finally, responses with more than one theme were coded for each relevant theme.

## 3. Results

### 3.1. Demographic Characteristics

A total of 477 RDNs responded to the email invitation, yielding a 9.57% response rate. Participants were primarily women (*n* = 457; 95.8%), had an average age of 43.96 (±15.04), and were predominantly White/Non-Hispanic (*n* = 443; 92.9%), with the majority possessing a Master’s degree (*n* = 264; 55.3%). The mean body mass index (BMI) was 24.77 kg/m^2^; 2.5% were classified as underweight, 61.5% were classified as normal weight, 22.2% were classified as overweight, and 13.8% were classified in the obesity category. See Table 1 for the complete descriptive report.

### 3.2. Health Changes

**Figure 1:**
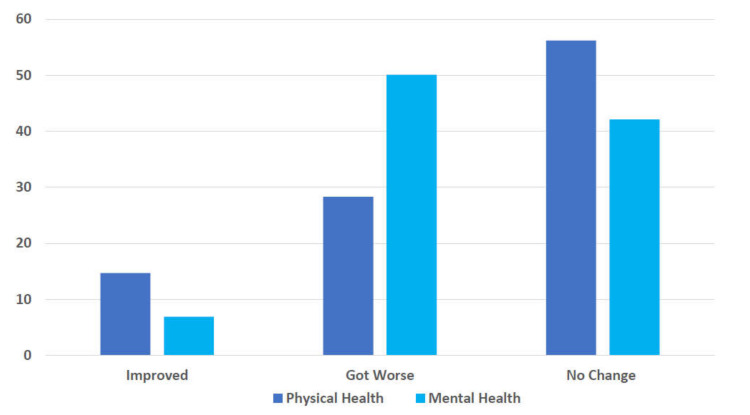
Percent of RDNs who perceived physical and mental health changes during the COVID-19 pandemic. (*n* = 473).

One survey question asked, “How do you think your physical health changed during the COVID-19 pandemic period?” A total of 56.2% of respondents reported no change in physical health during the pandemic, and 28.3% reported a decline. Another survey question asked, “How do you think your mental health changed during the COVID-19 pandemic period?” Half of the respondents indicated that their mental health worsened during the pandemic, and 42% reported no change (see Figure 1).

### 3.3. Weight Change

**Figure 2:**
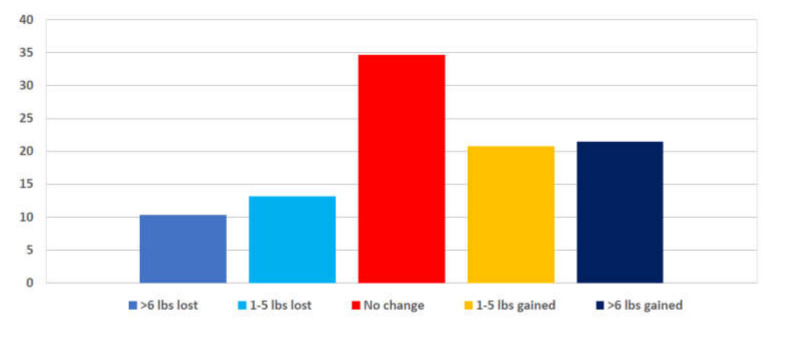
Percent of RDNs who self-reported weight change during the height of the COVID-19 pandemic. (*n* = 450; individuals who reported pregnancy were excluded from analysis).

Respondents were asked to report any weight change they experienced due to the pandemic. Of the sample, 68.5% of RDNs reported no weight change, 21.4% reported weight gain greater than 5 pounds, and 10.3% reported weight loss greater than 5 pounds. In addition, 75.5% of respondents indicated their weight change was unintentional (see Figure 2).

### 3.4. Changes in Eating Patterns

Survey questions asked respondents a variety of questions related to their eating patterns, such as “During the height of the COVID-19 pandemic in your area, did your appetite change?” Most respondents reported no changes in eating behaviors across the categories. Specifically, no changes in appetite (65.8%), portion sizes (73.2%), caffeine intake (67.5%), alcohol intake (54.7%), fruit intake (75.9%), vegetable intake (70.0%), fast food or take out (49.9%), or snacking/grazing (45.7%) were reported. In contrast, roughly half increased snacking/grazing (44.45%) and increased meal preparation at home (53.7%) (see Table 2).

### 3.5. Physical Activity

Survey questions asked respondents to assess current physical activity levels by asking, “On average, how many minutes/hours a day are you currently engaging in moderate-intensity activities?” Of the respondents, 29.1% of RDNs reported engaging in 30–45 min daily, 27.0% engaging in 15–30 min daily, and 19.1% engaged in <15 min daily (see Table 3). 

### 3.6. Psychological Factors

A validated scale, GAD-7, was used to measure anxiety among the survey respondents. A total of 53% of respondents met the criteria for minimal anxiety, 31% for mild anxiety, 9% for moderate anxiety, and 7% for severe anxiety. When asked about general health conditions, it is worth noting that 70.2% reported no anxiety, while 28.9% indicated a health professional had informed them that they had anxiety. 

**Figure 3:**
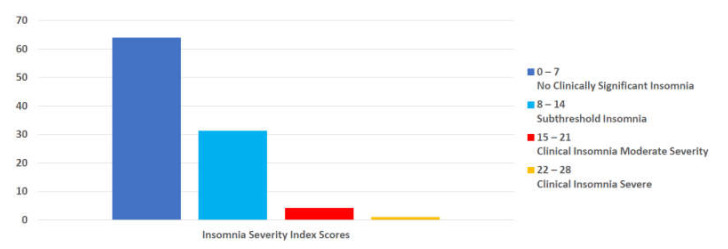
Percent of participants reporting sleep disturbances by Insomnia Severity Index (ISI) category. (*n* = 462).

**Figure 4:**
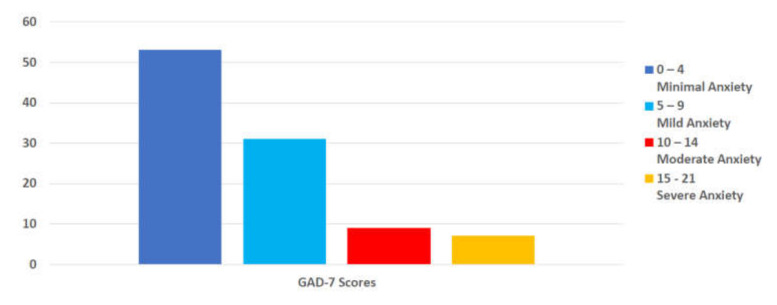
Percent of participants reporting anxiety by Generalized Anxiety Disorder (GAD-7) category. (*n* = 456).

A validated scale, ISI, was used to measure insomnia among the survey respondents. A total of 63.9% of respondents’ scores indicated no clinically significant insomnia, and 31.2% met the criteria for subthreshold insomnia. Moderate-severity and severe insomnia were reported by 4.1% of respondents and 0.9%, respectively. Of note, when asked about general health conditions, 89.3% reported no sleep problems, and only 9.9% indicated a health professional had informed them that they had sleep problems (see Figure 3 and Figure 4).

Additionally, relationships between the GAD-7 and ISI scales were examined using Pearson correlation analysis. It was found that those with higher GAD-7 scores were positively correlated with higher ISI scores (r = 0.393, *p* < 0.001), indicating that those who reported higher anxiety also reported having higher levels of insomnia.

### 3.7. Family/Caretaking Responsibilities

Most respondents (69.6%) reported that the pandemic did not significantly affect family or caretaking responsibilities. Although 26.4% noted a significant impact, most respondents (82.3%) did not think these changes resulted in significant food intake/behavior changes.

### 3.8. Professional Practice

Over half of respondents (55.3%) had a master’s degree, and the most common practice settings were hospitals (20.3%), outpatient clinics (17.4%), or long-term care facilities (15.5%). A total of 65.0% worked full-time, with 65.6% working typical weekday hours. While 34.4% reported significant changes in responsibilities due to the pandemic, over half (55.3%) experienced no or only slight changes in work responsibilities. In addition, 56.8% reported their professional training as an RDN equipped them to handle pandemic-related changes at work. Of the RDNs, 75.5% and 62.3%, respectively, reported their professional training equipped them with the skills needed to maintain healthy eating behaviors and physical activity. However, of note, 17.6% and 25.2%, respectively, reported that despite having the appropriate skills, the pandemic made it challenging to put healthy eating and physical activity skills into practice consistently.

### 3.9. Qualitative Responses

Of 477 participants, 255 answered open-ended Q1, yielding a 53% response rate. Two-hundred and three participants provided Q2 responses, yielding a 42.6% response rate. Participant responses from Q1 were coded into eight themes that emerged: reduced physical activity, increased physical activity, unfavorable eating habits, improved eating habits (with subthemes of intentional diet/weight changes), change in alcohol use, job/family/schedule disruption, mental health, and health/medical conditions. Q2 invited participants to further explain their responses to Q1. Over 40% indicated that reduced physical activity was the primary reason for weight change, followed by mental health concerns at 25.5%. In contrast to these negative health effects, the qualitative data also reveals positive health adaptations, such as increased physical activity and improvements in eating habits, which are data not commonly seen during the COVID era of research. See Table 4 for themes, frequencies, definitions, and illustrative supportive quotes.

## 4. Discussion

The COVID-19 pandemic triggered a global health crisis that sparked a cascade of abrupt life and work adaptations with numerous health and professional consequences. During the COVID-19 pandemic, healthcare workers were undoubtedly susceptible to increased levels of stress, anxiety, and emotional fatigue [1,2,9]. Studying the pandemic experience among RDNs presents a unique opportunity to represent RDNs in emerging research and bring to light new information on the influence of stress and life disruption on weight, health behaviors, and professional practices. 

While dietitians are often asked to advise the public about healthy weight or weight management, this is the first questionnaire study to our knowledge that explored RDNs’ perceptions relating to maintaining their own health behaviors during the COVID-19 pandemic. This study requested that RDNs self-reflect on personal health behaviors and health preservation abilities. Key results indicated that RDNs’ specialized knowledge and experience conferred some level of protection from pandemic-related disruptions in healthy eating patterns and unintentional weight gain compared to other healthcare workers and the general population. RDNs are not immune to work and life stressors, and while clear causal relationships cannot be established, this study suggests that professional training and skillsets may equip RDNs with strategies to adapt to changing circumstances. 

During the pandemic, RDNs’ work and life experiences were subject to changes based on each of his/her distinctive roles. Some RDNs in the clinical setting were charged with COVID-19-specific role changes, such as identifying and addressing malnutrition in treating and preventing further adverse health outcomes from COVID-19 infection [26]. For others in private practice or outpatient settings, the pandemic required innovative nutrition counseling to be delivered via telehealth, introducing opportunities to provide counseling virtually but requiring an abrupt change in care modality. Although this study indicates that no or only slight changes in work responsibilities occurred for many (55.4%), just over one-third of the respondents experienced significant alterations in job responsibilities, presenting additional challenges that could affect eating behaviors or physical or mental health. 

Self-reported weight gain (weight gain >5 lbs.) was noted in 21.4% of study respondents. This was a significantly lower weight gain compared to reports of the general population [27]. In a review of the American Psychological Association’s Stress in America report (2021), 61% of surveyed adults experienced undesired weight changes since the start of the pandemic, averaging about 29 pounds [28]. The qualitative data also lend further insight into RDN health behaviors and weight change. The top reasons reported by respondents about weight change were reduced physical activity and mental health; however, the next two responses were positive behaviors—improved eating habits and increased physical activity—thereby potentially supporting why this RDN sample may have experienced less weight gain than their counterparts. While we acknowledge that weight status and weight change alone are not definitive indicators of health, nor simply being an RDN as the only protective factor against weight fluctuations, the combination of weight stability and the preservation of healthy eating and lifestyle-related behaviors among our sample of RDNs during stressful periods is noteworthy. 

Trends noted in both eating and physical activity habits were demonstrated by 75.5% and 62.3% of participants, respectively, who reported that their RDN professional training equipped them with the skills needed to maintain these habits. Regarding physical activity, 42.6% of respondents maintained physical activity levels of at least 30 min daily, compared to other studies, which reported decreases of up to 46% for moderate-intensity activity and ranges of 32–43% for overall physical activity [29,30]. In addition, while 40.8% of Q1 respondents indicated reduced physical activity as a probable reason for weight change, 17.6% of Q1 respondents indicated they had greater frequency, intensity, and/or duration of exercise, which had a beneficial impact on weight control. Although recent studies have reported significant pandemic-related changes in weight and increased snacking among large segments of the population, this study’s participants did not exhibit those behaviors to similar extremes [27,31,32,33]. Qualitative data also support this, as 17.6% of Q1 respondents indicated improved eating habits. 

Regarding psychological factors, GAD-7 results revealed that while 16% of participants met criteria for moderate to severe anxiety, over 80% of the respondents did not. In comparison, the National Center for Health Statistics (NCHS) Household Pulse Survey conducted during the same frame as our study revealed that, on average, 35.8% reported significant anxiety symptoms based on the GAD-2, an abbreviated version of GAD-7, with a range of 28.1–41.2% when separated by race/ethnic groups [34]. When anxiety was assessed with a broadened view, including qualitative data responses, the general theme of mental health was identified as the second most frequent reason for weight change in Q1 (25.5%), which parallels the percent of GAD-7 results of probable anxiety disorder. Additionally, mental health was also linked to responses that described job/family/lifestyle disruptions (8%) and unfavorable eating habits (8%). Overall, these findings may suggest that RDNs fared better and with less anxiety when compared with the general population; however, they were not immune to these psychological stressors during the pandemic. 

According to the ISI, most RDNs in our study experienced no significant levels of insomnia, with less than 5% meeting the criteria for moderate or severe insomnia. In comparison, a 2021 systematic review and meta-analysis found a pooled estimated prevalence of sleep problems to be 31% among healthcare professionals and 18% among the general population [35]. Other studies that looked solely at healthcare workers found more sleep disturbances in that population than non-healthcare professionals, with one study finding nearly 96% of surveyed healthcare workers reporting poor sleep and 30% reporting moderate or severe insomnia [36]. It is possible that the regular work schedule and routine endorsed by most of the respondents at the time of the questionnaire administration reduced the risk of insomnia. Poor sleep quality is also frequently associated with weight gain, and the low rates of insomnia reported by our RDN respondents may have contributed to weight stability in RDNs during the pandemic [37].

Three-quarters of respondents reported that their professional training equipped them adequately for maintaining healthy eating habits during the pandemic, and nearly two-thirds indicated that professional training left them adequately equipped to maintain physical activity habits. This finding is not surprising, as the pathway to becoming an RDN provides a solid foundation in many areas of nutrition, health promotion, obesity prevention and treatment, lifestyle, behavioral changes, and adapting nutritional needs for a multitude of lifestyle needs or medical conditions. COVID-19 restrictions may have afforded RDNs with more time to spend on activities or commit to habits they value, which, by nature of being an RDN, may be assumed to be health related. However, knowledge, practice, and understanding of the value of health do not guarantee perfect adherence. Stressful circumstances can present challenges to even those well-equipped, as indicated by the 17.6% of respondents who indicated that pandemic-related circumstances made it difficult to consistently put healthy eating into practice, and 25.2% reported that pandemic circumstances made it difficult to meet goals for physical activity. Thus, additional support may be warranted, even for those who are well-trained in nutrition. 

### Strengths and Limitations

There were several strengths to this study. First, to our knowledge, this is the first study that captures experiences of RDNs across the United States during the COVID-19 pandemic peak of winter 2021 and the impact of pandemic-related changes on weight, eating behaviors, physical activity, and psychological factors. With well over 100,000 RDNs in the United States, it is essential for their experiences to be understood, especially those relating to lifestyle, physical, and mental health [38]. Given RDNs are at the forefront of health promotion, medical nutrition therapy, and assisting patients/clients with lifestyle changes, ensuring they are as healthy as possible helps in this role. Additionally, the many free text comments received provided rich qualitative data, further adding dimension and context to the findings. Finally, the responses to the questions regarding the professional preparation of RDNs and the relationship to personal adaptations made during the pandemic afforded an opportunity to gain some perspective on professional training and potentially personal health impact. 

This study is not without its limitations. First, the data presented represent self-reported measures by a subset of RDNs at one-time point, and therefore, generalizability to other groups is limited. The research team developed the quantitative assessment questions that gathered data on eating patterns, physical activity, and perceived physical and mental health data; these questions lacked validation. Next, responses to the GAD-7 and ISI were not verified by a clinical diagnosis. Additionally, most survey respondents were working full-time and had a regular weekday schedule, and therefore, those RDNs working erratic schedules or subject to other significant stressful work scenarios may have been underrepresented. Other factors that were not controlled for or not captured by this research must also be considered. Finally, the demographic breakdown of our RDN respondent pool lacked diversity, which may also limit generalizability [39].

## 5. Conclusions

These findings suggest that the professional practice skills of RDNs may have conferred some personal health benefits, as evidenced by smaller weight gains, the maintenance of healthy habits, and smaller numbers reporting psychological effects compared to the general population and other health professionals, thereby limiting the impact of pandemic-induced disruptions. However, any changes that may disrupt eating patterns, physical activity, or overall lifestyle may impact overall health if sustained long-term. Therefore, future research that focuses on supporting the healthful eating habits of all individuals beyond the acute pandemic effects is essential. In addition, implementing ongoing health promotion strategies to ensure these dietary changes are not permanent may benefit the health and wellness of all populations.

## Figures and Tables

**Table 1:** Demographic characteristics of a subsample of Registered Dietitian Nutritionists (RDN) participants. (*n* = 477).

Variables	Total (*n* = 477)
**Gender**	***n*** **(%)**
Male, *n* (%)	16 (3.4)
Female, *n* (%)	457 (95.8)
Age, mean (SD), years old	43.96 (±15.04)
**Race**	***n*** **(%)**
Black/African American	8 (1.7)
Latinx/Hispanic	6 (1.3)
White/Non-Hispanic	443 (92.9)
Asian/Pacific Islander	9 (19)
Multi-racial/Mixed ethnicities	5 (1.0)
Other	2 (0.4)
**Body Mass Index** (*n* = 448)	
BMI, mean (S.D.)	24.77 (±4.99)
Underweight	11 (2.5)
Normal weight	271 (61.5)
Overweight	98 (22.2)
Obesity	61 (13.8)
**Highest education level**	***n*** **(%)**
Some college or trade school	1 (0.2)
Associates or Technical Degree	2 (0.4)
Bachelor’s Degree	181 (37.9)
Master’s Degree	264 (55.3)
Doctoral Degree (e.g., J.D., MD, PhD)	25 (5.2)
**Primary Practice Setting**	***n*** **(%)**
Academic Institution	39 (8.2)
Community	26 (5.5)
Foodservice	13 (2.7)
Hospital	97 (20.3)
Not Currently Working	41 (8.6)
Long-Term Care Facility	74 (15.5)
Out-Patient Clinic	83 (17.4)
Private Practice (Inside your home)	34 (7.1)
Private Practice (Outside your home)	24 (5)
Retail	3 (0.6)
Other	39 (8.2)
**Employment Status**	***n*** **(%)**
Full-Time Student	3 (0.6)
Part-time	98 (20.5)
Full-time (>35 h per week)	310 (65.0)
Retired	25 (5.2)
Unemployed Due to COVID	8 (1.7)
Unemployed not Related to COVID	10 (2.1)
Not Working Due to Disability	2 (0.4)
Other	17 (3.6)
Number of Years in Practice, mean (SD), y	17.39 (±14.52)
**Typical Workday**	***n*** **(%)**
Flexible hours (make your own schedule)	83 (17.4)
Irregular hours (i.e., changes day to day or week to week)	17 (3.6)
Per diem (as needed) hours	16 (3.4)
Weekday hours	313 (65.6)
I am not currently working	44 (9.2)

**Table 2:** Eating pattern changes during the height of the COVID-19 pandemic among RDNs. *n* (%).

	Less/Decrease	No Change	More/Increase
Home meal preparation frequency	38 (8.0)	171 (35.8)	256 (53.7)
Snacking/grazing	35 (7.3)	218 (45.7)	212 (44.4)
Alcoholic beverage intake	61 (12.8)	261 (54.7)	143 (30.0)
Caffeinated beverage intake	30 (6.3)	322 (67.5)	113 (23.7)
Fast food or take out consumption	128 (26.8)	238 (49.9)	99 (20.8)
Appetite	54 (11.3)	314 (65.8)	97 (20.3)
Daily vegetable intake	45 (9.4)	334 (70.0)	86 (18.0)
Food portion sizes	49 (10.3)	349 (73.2)	67 (14.0)
Daily fruit intake	38 (8.0)	362 (75.9)	65 (13.6)

**Table 3:** Physical activity averages with moderate-intensity activities among RDNs during the COVID-19 pandemic. *n* (%).

Minutes/Hours/Day	*n* (%)
<15 min	91 (19.1)
15–30 min	129 (27.0)
30–45 min	139 (29.1)
45 min–1 h	82 (17.2)
>1 h	30 (6.3)

**Table 4:** Themes, response frequencies, definitions, and illustrative supportive quotes (from Q2) to open-ended question Q1: “If your weight changed during the height of the pandemic period, please describe why you think your weight changed” (*n* = 255).

Theme	Response Frequency	Definition	Illustrative Quotes
Reduced physical activity	*n* = 104, 40.8%	Decreased frequency, intensity and/or duration of exercising	“Due to limited home space and quarantine, I have given up continuing with favorite physical activities like yoga or group fitness classes, which I think has impacted my mental health and ability to handle small daily stressors.” “Not being able to go out to my exercise class got me out of habit of exercising and I watch what I eat less if I am NOT exercising. I am also more sedentary working remotely—I would move around more in the office.”
Mental health	*n* = 65, 25.5%	Increase in stress, anxiety, depression, emotional eating, boredom leading to an increase or decrease in food intake	“I have increased my work hours from ~50 to 60–65 h a week. My stress level has increased from an already stressful position, and I believe that this stress has contributed to me not truly caring or able to make sound decisions in healthful behaviors. I feel like I have been in “fight” mode for a year and truly not able to center enough to use my training on a personal level.” “In the very beginning of COVID (March-April (2019) I was terrified of COVID. This caused me to have severe panic attacks and severe anxiety. I was afraid to go to work. My mental health took a huge toll. I was doing my best to maintain physical activity and eating healthy. I lost a few pounds in the beginning due to my anxiety over the pandemic. Come June (2019), I adapted to the “new way of life” and have been fine ever since.” “The stress of the pandemic has been mentally debilitating. Working from home full time, virtual kindergarten and keeping up a household have destroyed my mental stability. Using tools that I know can help me (and my family feel better has been what keeps us going). Cooking ourselves, eating healthy food, exercising regularly and for myself, really not drinking alcohol have been a help but honestly I’m hanging on by a thread.”
Unfavorable eating habits	*n* = 49, 19.2%	Unintentional, detrimental shifts or modifications in dietary patterns	“Food eating habits changed slightly for me in respect to the quality of food. I didn’t necessarily eat more but indulged in “higher energy foods”! For example, using more butter, more chocolate. For my family, I did purchase more processed foods than usual as they seemed to want to “graze” more! For example, ice cream, potato chips. Typically, these were treats that were brought home occasionally.” “COVID-19 has made me lazy. I don’t want to cook anymore, and I look for quick cooking instead (pizza, rice, burgers, frozen foods). I also snack way more often than I ever did before. I used to never buy snack foods and now I have a favorite brand of chips!!! That has never happened before.”
Improved eating habits	*n* = 45, 17.6%	Intentional or unintentional resetting or correction of dietary patterns	“Made a conscious effort to eat b/l/d and snacks on a schedule that reflected our pre-pandemic schedule; also made a pact not to bake desserts/breads. Focused on making fun meals, and not increasing desserts/snack foods.—instead of shopping 3x/wk at different stores, shopped every 10 days at 1 store, decreased variety of fresh f/v and variety of specialty items that I would normally get at the Asian, Mid East, Indian stores.”
Subtheme: Intentional weight/diet changes	*n* = 19, 7.5%	Actively focusing on improving diet to promote healthy weight	“Initially at the start of quarantine—I took the opportunity to do a pantry/fridge/freezer clean out to limit food waste and having to leave the house out of fear. As restrictions eased, cases went down, I became a smarter grocery shopper—from planning, the grocery list, to shopping, more efficient and purposeful.”
Increased physical activity	*n* = 45, 17.6%	Greater frequency, intensity and/or duration of exercising	“Due to gyms being closed, I began yoga at the start of quarantine. A year later, I am still loving the practice. It helps me relax and destress. We created a home gym in the basement as well.” “More leisure time spent being physically active outside.”
Job/family/schedule disruptions	*n* = 42, 16.5%	Changes from usual work or home/family routines significant enough to impact diet, physical activity, or weight	“My job becoming virtual has led to me moving around less... Overall meal balance decreased with an increase in ordering takeout because I was tired often after work and didn’t want to cook. Fatigued by the end of the day often due to looking at the computer screen for 8 h when it used to be only a few hours before with more in person interaction. This has made it difficult to maintain a healthy balance with exercise.” “My main problem with my family’s food intake is the kids desire for constant snacks being home all day or with boredom they’d think they were hungry. It was also more difficult to get grocery shopping scheduled as I typically did this while they were in school. They are in virtual school, and I did not want to take 2 kids out to the grocery store. Some home delivery services were too expensive. I consider myself privileged to have more flexibility and resources and felt bad imagining people in more difficult circumstances.” “At the beginning of the pandemic dietitians in hospital were left to figure things out for themselves to create safe working habits and spaces with slow guidance from hospital and zero from ADA. Worry early on about bringing COVID home to vulnerable family members added stress/poor sleeping and eating habits.”
Health/medical conditions	*n* = 34, 13.3%	Pregnancy or acute/chronic conditions	“At first I was affected as just as COVID was hitting, I had ear surgery and was not allowed to even walk on treadmill for 8 weeks. I gained weight from stress eating. Then I injured my right arm, and it took about 4–6 weeks to heal.” “The biggest challenge for exercise is having a baby/ toddler. I don’t do enough dedicated exercise, but I am active with a toddler.”
Change in alcohol use	*n* = 18, 7%	Increased or decreased consumption of beer, wine, hard liquor	“I was always starving by dinner so I would eat too much. Combine that with too much wine to cope with the stress- and it’s no wonder I gained so much weight. I could almost watch the weight gain … and I felt completely powerless to stop it.” “COVID affected my eating and drinking habits. I was “comfort eating” at times and definitely drinking more wine. There was nothing else to do! I gained 5–10 lbs.”

## Data Availability

The data presented in this study are available on request from the corresponding author. The data are not publicly available due to privacy.

## References

[1] Di Renzo L., Gualtieri P., Pivari F., Soldati L., Attinà A., Cinelli G., Leggeri C., Caparello G., Barrea L., Scerbo F. (2020). Eating habits and lifestyle changes during COVID-19 lockdown: An Italian survey. J. Transl. Med..

[2] Greenberg N. (2020). Mental health of health-care workers in the COVID-19 era. Nat. Rev. Nephrol..

[3] Barello S., Palamenghi L., Graffigna G. (2020). Burnout and somatic symptoms among frontline healthcare professionals at the peak of the Italian COVID-19 pandemic. Psychiatry Res..

[4] Risk for COVID-19 Infection, Hospitalization, and Death by Race/Ethnicity. https://www.cdc.gov/coronavirus/2019-ncov/covid-data/investigations-discovery/hospitalization-death-by-race-ethnicity.html.

[5] Kazmierski K.F.M., Gillespie M.L., Kuo S., Zurita T., Felix D., Rao U. (2021). Stress-Induced Eating Among Racial/Ethnic Groups in the United States: A Systematic Review. J. Racial Ethn. Health Disparities.

[6] Khaled K., Tsofliou F., Hundley V., Helmreich R., Almilaji O. (2020). Perceived stress and diet quality in women of reproductive age: A systematic review and meta-analysis. Nutr. J..

[7] Neill E., Meyer D., Toh W.L., Van Rheenen T.E., Phillipou A., Tan E.J., Rossell S.L. (2020). Alcohol use in Australia during the early days of the COVID-19 pandemic: Initial results from the COLLATE project. Psychiatry Clin. Neurosci..

[8] Puhl R.M., Lessard L.M., Larson N., Eisenberg M.E., Neumark-Stzainer D. (2020). Weight Stigma as a Predictor of Distress and Maladaptive Eating Behaviors During COVID-19: Longitudinal Findings from the EAT Study. Ann. Behav. Med..

[9] Carroll N., Sadowski A., Laila A., Hruska V., Nixon M., Ma D., Haines J., on behalf of the Guelph Family Health Study (2020). The Impact of COVID-19 on Health Behavior, Stress, Financial and Food Security among Middle to High Income Canadian Families with Young Children. Nutrients.

[10] Academy of Nutrition and Dietetics Coronavirus (COVID-19) Professional Resource Hub. https://www.eatrightpro.org/coronavirus-resources.

[11] De Costa Matos R.A., de Cássia Coelho de Almeida Akutsu R., Zandonadi R.P., Rocha A., Botelho R.B.A. (2020). Wellbeing at Work before and during the SARS-COV-2 Pandemic: A Brazilian Nationwide Study among Dietitians. Int. J. Environ. Res. Public Health.

[12] Heitman K. COVID-19 Stories and Experiences; Food & Nutrition Magazine. https://foodandnutrition.org/covid-19/covid-19-stories-and-experiences.

[13] Rozga M., Handu D., Kelley K., Jimenez E.Y., Martin H., Schofield M., Steiber A. (2021). Telehealth During the COVID-19 Pandemic: A Cross-Sectional Survey of Registered Dietitian Nutritionists. J. Acad. Nutr. Diet..

[14] Epel E., Lapidus R., McEwen B., Brownell K. (2001). Stress may add bite to appetite in women: A laboratory study of stress-induced cortisol and eating behavior. Psychoneuroendocrinology.

[15] O’Connor D.B., Jones F., Conner M., McMillan B., Ferguson E. (2008). Effects of daily hassles and eating style on eating behavior. Health Psychol..

[16] Donnelly R., Keller H. (2021). Challenges Providing Nutrition Care during the COVID-19 Pandemic: Canadian Dietitian Perspectives. J. Nutr. Health Aging.

[17] Naja F., Radwan H., Ismail L.C., Hashim M., Rida W.H., Abu Qiyas S., Bou-Karroum K., Alameddine M. (2021). Practices and resilience of dieticians during the COVID-19 pandemic: A national survey in the United Arab Emirates. Hum. Resour. Health.

[18] Qualtrics (2021). Computer Software.

[19] Spitzer R.L., Kroenke K., Williams J.B.W., Löwe B. (2006). A Brief Measure for Assessing Generalized Anxiety Disorder: The GAD-7. Arch. Intern. Med..

[20] Bastien C.H., Vallieres A., Morin C.M. (2001). Validation of the Insomnia Severity Index as an outcome measure for insomnia research. Sleep Med..

[21] (2020). Statistical Package for the Social Sciences—SPSS (Version 24).

[22] Löwe B., Decker O., Müller S., Brähler E., Schellberg D., Herzog W., Herzberg P.Y. (2008). Validation and Standardization of the Generalized Anxiety Disorder Screener (GAD-7) in the General Population. Med. Care.

[23] Morin C.M., Belleville G., Bélanger L., Ivers H. (2011). The Insomnia Severity Index: Psychometric Indicators to Detect Insomnia Cases and Evaluate Treatment Response. Sleep.

[24] Vaismoradi M., Turunen H., Bondas T. (2013). Content analysis and thematic analysis: Implications for conducting a qualitative descriptive study. Nurs. Health Sci..

[25] (2021). ATLAS.ti.

[26] Handu D., Moloney L., Rozga M., Cheng F.W. (2021). Malnutrition Care During the COVID-19 Pandemic: Considerations for Registered Dietitian Nutritionists. J. Acad. Nutr. Diet..

[27] Chew H., Lopez V. (2021). Global Impact of COVID-19 on Weight and Weight-Related Behaviors in the Adult Population: A Scoping Review. Int. J. Environ. Res. Public Health.

[28] American Psychological Association STRESS IN AMERICA™ One Year Later, A New Wave of Pandemic Health Concerns. https://www.apa.org/news/press/releases/stress/2021/one-year-pandemic-stress.

[29] Meyer J., McDowell C., Lansing J., Brower C., Smith L., Tully M., Herring M. (2020). Changes in Physical Activity and Sedentary Behavior in Response to COVID-19 and Their Associations with Mental Health in 3052 US Adults. Int. J. Environ. Res. Public Health.

[30] Dunton G.F., Wang S.D., Do B., Courtney J. (2020). Early effects of the COVID-19 pandemic on physical activity locations and behaviors in adults living in the United States. Prev. Med. Rep..

[31] Robinson E., Boyland E., Chisholm A., Harrold J., Maloney N.G., Marty L., Mead B.R., Noonan R., Hardman C.A. (2021). Obesity, eating behavior and physical activity during COVID-19 lockdown: A study of UK adults. Appetite.

[32] Park S., Lee S.H., Yaroch A.L., Blanck H.M. (2022). Reported Changes in Eating Habits Related to Less Healthy Foods and Beverages during the COVID-19 Pandemic among US Adults. Nutrients.

[33] Lombardo M., Guseva E., Perrone M.A., Müller A., Rizzo G., Storz M.A. (2021). Changes in Eating Habits and Physical Activity after COVID-19 Pandemic Lockdowns in Italy. Nutrients.

[34] Centers for Disease Control and Prevention Health Care Access, Telemedicine, and Mental Health. https://www.cdc.gov/nchs/covid19/health-care-access-and-mental-health.htm.

[35] Alimoradi Z., Broström A., Tsang H.W., Griffiths M.D., Haghayegh S., Ohayon M.M., Lin C.-Y., Pakpour A.H. (2021). Sleep problems during COVID-19 pandemic and its’ association to psychological distress: A systematic review and meta-analysis. EClinicalMedicine.

[36] Stewart N.H., Koza A., Dhaon S., Shoushtari C., Martinez M., Arora V.M. (2021). Sleep Disturbances in Frontline Health Care Workers During the COVID-19 Pandemic: Social Media Survey Study. J. Med Internet Res..

[37] Cooper C.B., Neufeld E.V., Dolezal B.A., Martin J.L. (2018). Sleep deprivation and obesity in adults: A brief narrative review. BMJ Open Sport Exerc. Med..

[38] Commission on Dietetic Registration. Registry Statistics. https://www.cdrnet.org/registry-statistics.

[39] Rogers D. (2021). Report on the Academy/Commission on Dietetic Registration 2020 Needs Satisfaction Survey. J. Acad. Nutr. Diet..

